# Dry Eye Disease: Bridging Systemic Inflammation, Ocular Surface Biology, and Clinical Innovation

**DOI:** 10.3390/biomedicines14030684

**Published:** 2026-03-17

**Authors:** Liat Gantz

**Affiliations:** Department of Optometry and Vision Science, Jerusalem Multidisciplinary College, Jerusalem 9101001, Israel; liatg@jmc.ac.il

Dry eye disease (DED) is a prevalent and complex disease of the ocular surface [1], characterized by tear film and/or ocular surface instability, inflammation, ocular surface damage, tear film hyperosmolarity and neurosensory abnormalities [2] that negatively impact visual function and quality of life.

Despite major advances in diagnostic technology and therapeutic strategies, significant challenges persist in accurately identifying reliable biomarkers, and delivering therapeutic care. Inter-individual variability and the absence of universally accepted disease endotypes limit both diagnostic precision and therapeutic targeting. DED is defined largely by clinical descriptors rather than by biologically stratified mechanisms. The present Special Issue of Biomedicines brings together eight original research studies and one systematic review that collectively reflect the evolving, multidisciplinary landscape of dry eye research.

These contributions suggest that DED should be conceptualized as a systemic–ocular interface condition shaped by inflammatory, biomechanical, neurosensory, and psychosocial determinants. This approach aligns with emerging precision-medicine paradigms.

A central theme emerging from this Special Issue is the growing recognition of DED as a condition that is closely linked to systemic inflammatory and metabolic pathways. 

Kabakci et al. demonstrate that the systemic inflammation response index and the C-reactive protein-to-albumin ratio obtained from blood tests are independently associated with both the presence and severity of dry eye disease in patients with Hashimoto’s thyroiditis-induced hypothyroidism. This demonstrates that dry eye disease is common in patients with Hashimoto’s thyroiditis-induced hypothyroidism, and the potential role of routine blood-derived inflammatory indices as accessible adjunctive biomarkers for improved detection and stratification of DED in this population [3].

Wang et al. examined how obstructive sleep apnea and its treatment may influence dry eye disease. Their study demonstrates that excessive positive airway pressure mask leakage, indicative of suboptimal therapy, is associated with reduced tear production and optic nerve structural markers, highlighting a plausible mechanism linking sleep-disordered breathing, ocular surface disease, and glaucoma. These findings underscore the need for closer interdisciplinary collaboration and suggest that optimizing airway mask delivery may be critical for mitigating ocular morbidity in patients with obstructive sleep apnea [4].

Ifrah & Darwish describe a substantially higher prevalence and severity of objective MGD signs in keratoconus patients (33 eyes) compared with healthy controls (27 eyes) in the Palestinian Authority, highlighting the importance of routine ocular surface assessment and integrated management in this population [5]. Given that keratoconus patients are often corrected with contact lenses, and its corneal ectatic nature, the causative mechanism for dry eye in keratoconus patients remains to be elucidated.

Zhang et al. provide large-scale epidemiological evidence from the All-of-Us Research Program, demonstrating that obstructive sleep apnea is independently associated with higher prevalence of both DED (19% compared with 6% in controls) and meibomian gland dysfunction (3% compared with 1% in controls) in a diverse U.S. population. These findings emphasize the importance of routine ocular surface assessment in these patients [6]. Future studies should examine the possible links between the conditions.

In a comparative study of 38 non-DED and 34 DED patients, significant differences were observed in multiple corneal biomechanical parameters, including A1 deflection area, A1 delta arc length, A2 deformation amplitude, and whole eye movement. These biomechanical changes were significantly correlated with tear meniscus height among DED patients, suggesting that dry eye disease, and particularly reduced tear volume, may meaningfully influence corneal biomechanical behavior [7]. Whether biomechanical differences affect refractive measurements, surgical planning, or long-term corneal stability in DED populations requires larger, longitudinal investigations. Without such data, biomechanical alterations risk remaining physiologically interesting yet clinically ambiguous observations with their translational significance yet to be established. 

The therapeutic studies in this issue highlight the growing shift toward biologic and device-based interventions. 

A systematic review and meta-analysis of 17 comparative studies comparing the clinical efficacy of autologous serum, platelet-rich plasma and artificial tears in the treatment of DED shows that autologous serum and platelet-rich plasma demonstrate greater improvements in symptoms, tear break up time and Schirmer test scores compared to artificial tears. Injectable platelet-rich plasma shows a promising trend toward better outcomes than autologous serum, though standardized, long-term trials are necessary to enable direct comparisons of efficacy and safety [8]. Additionally, cost, accessibility, and regulatory considerations must be addressed before widespread implementation.

Tan et al. [8] present a randomized pilot trial demonstrating that a prototype electronic heating device is safe and effective for at-home management of MGD, with improvements in meibomian gland function and patient-reported comfort comparable or superior to standard warm compress therapy, each tested in a cohort of 10 participants. This study reflects the growing shift toward device-based, standardized home therapies aimed at improving treatment adherence and reproducibility. This encouraging early-phase trial warrants a longer evaluation with a larger sample size for meaningful integration into clinical guidelines.

Repetitive magnetic stimulation was evaluated as a three-month therapeutic intervention for moderate-to-severe dry eye disease in 22 participants, compared with a placebo-treated control group. No between-group differences were observed in visual acuity, intraocular pressure, or Schirmer test results and symptom scores improved in both groups. However, significant improvements in tear film stability and corneal surface health were observed only in the treatment group [9]. While promising, this treatment modality warrants further evaluation in larger cohorts and across clearly defined severity subgroups to better determine its efficacy and appropriate clinical indications.

Together, these studies underscore the importance of interdisciplinary collaboration between ophthalmology, optometry, psychology, pulmonology, and internal medicine in the holistic management of DED.

Beyond structural and epidemiological perspectives, the psychosocial dimension of dry eye is addressed in a study by Gantz et al. [10], which demonstrates that greater loss of meibomian glands in the lower eyelids is significantly associated with reduced emotional and functional scores in keratoconic patients with dry eye. This work highlights that objective signs alone cannot fully capture the lived experience of ocular surface disease and the importance of incorporating patient-reported outcome measures into routine clinical assessment. It further reinforces that the ocular surface health of keratoconic patients should be regularly assessed. 

The diagnosis of dry eye is symptom-driven, and the recently modified TFOS DEWS III [2] definition places greater emphasis on patient-reported symptoms, underscoring their central role in both classification and clinical decision-making.

Collectively, the articles in this Special Issue illustrate the multifactorial nature of dry eye disease, integrating systemic inflammation, sleep obstruction, other corneal disorders, corneal biomechanics, quality of life, and therapeutic innovation into a coherent research framework. These studies demonstrate the individualized nature of DED, considering overlapping phenotypes requiring personalized diagnostic and management strategies.

The convergence of systemic biomarkers, advanced imaging and therapeutics and patient-reported outcomes highlights the need for harmonized and standardized disease protocols. Future research directions emerging from this collection include the validation of systemic inflammatory biomarkers in diverse populations, longitudinal assessment of corneal biomechanical changes in DED, harmonization of biologic therapy protocols, and integration of digital and device-based therapies into evidence-based care pathways. Equally important is the continued emphasis on patient-centered outcomes, ensuring that clinical advances translate into meaningful improvements in daily functioning and well-being.

Multicenter prospective clinical trials using standardized protocols will advance clinical understanding and implementation to the next stages.

As Guest Editor, I would like to express my sincere gratitude to all contributing authors for their high-quality scientific work, to the reviewers for their thoughtful and constructive evaluations, and to the Biomedicines editorial team for their professional and dedicated support throughout the publication process. Special Issues such as this one will hopefully serve as a valuable reference for clinicians and researchers alike, fostering continued innovation and collaboration in the evolving field of dry eye disease.

## Data Availability

No new data were created or analyzed in this editorial.
